# Do Older Adults Select Appropriate Motor Strategies in a Stepping-Down Paradigm?

**DOI:** 10.3389/fphys.2018.01419

**Published:** 2018-10-12

**Authors:** Nick Kluft, Sjoerd M. Bruijn, Jaap H. van Dieën, Mirjam Pijnappels

**Affiliations:** Department of Human Movement Sciences, Vrije Universiteit Amsterdam, Amsterdam, Netherlands

**Keywords:** step descent, old age, degree of misjudgment, decision making, self-perception, falls, locomotion, perturbation

## Abstract

Selecting motor strategies in daily life tasks requires a perception of the task requirements as well as of one's own physical abilities. Age-related cognitive and physical changes may affect these perceptions. This might entail that some older adults select inappropriate movement strategies when confronted with daily-life motor tasks, which could lead to balance loss or falls. We investigated whether older adults select motor strategies in accordance with their actual physical ability. Twenty-one older adults were subjected to a stepping down paradigm, in which full-body kinematics of selected and reactive behavior were recorded. Stepping down from a curb can be done with either (1) a relatively low effort but more balance threatening heel landing, or (2) a more controlled but more demanding toe landing. The probability of selecting a toe landing grows with an increase in curb height. We determined the curb height at which participants switched from heel to toe landing during expected stepping down over different heights as an indicator of their perceived ability. During an unexpected step down trial, participants encountered a step down of 0.1 m earlier than expected, because part of the walkway was removed and covered by a black cloth. We evaluated participants' actual physical ability from the reactive behavior, with performance defined as the reduction in kinetic energy between the peak value after landing and the onset of the next step. To unravel whether the selected motor strategies corresponded with actual physical ability, the ability to recover from the unexpected step down was correlated to the height at which the participants switched movement strategy. The switching height was not correlated to the ability to recover from an unexpected step down (ρ = 0.034, *p* = 0.877). This finding suggests that older adults do not select their movement strategy in stepping down based on their actual abilities, or have an imprecise perception of their actual abilities. Future research should evaluate whether inappropriate motor strategy selection in a stepping down paradigm can explain accidental falls in older adults.

## Introduction

Moving through the environment requires integration of informational cues from the environment (Gibson, 1958). Combining these cues with the perception of one's physical abilities is essential for safe movement. However, 35 percent of the older adults experience a fall at least once a year (World Health Organization, 2007). Compared to young adults–with a fall incidence of 18 percent (Talbot et al., 2005)–older adults seem either vulnerable or reckless human beings, which puts into question older adults' ability to adapt their movement behavior to their actual physical abilities.

Appropriate perception of one's physical abilities would appear a necessity to avoid a mismatch between perceived and actual ability, but self-perception may be distorted in older adults, since even healthy aging is accompanied with a decline in cognitive capacities (Lustig et al., 2009; Segev-Jacubovski et al., 2011). Besides this cognitive decline, a decrease in physical abilities is observed as well (Vandervoort, 2002; Woollacott and Shumway-Cook, 2002), and continuous recalibration of perceived and actual abilities appears needed (Ellmers et al., 2018).

Two studies compared the actual ability to perform tasks to judgements of one's ability to perform these tasks (Butler et al., 2015; Kluft et al., 2016). When crossing narrow planks, almost one-third of the participating older adults showed risky behavior (Butler et al., 2015). Participants who chose a too narrow plank where more likely to fall in the upcoming year, showing the importance of such information for fall risk prediction models. In order to directly quantify the degree to which older adults misjudge their physical ability, we proposed a measure to evaluate the degree of misjudgment, and found that in most older adults perceived gait ability did not match their actual physical ability (Kluft et al., 2016). However, it is unclear whether this degree of misjudgment results in erroneous movement behavior.

A paradigm to investigate whether movement behavior is in alignment with movement ability, is motor strategy selection when stepping down a curb. It has been shown that there are two strategies of stepping down: a toe-landing strategy and a heel-landing strategy (Freedman and Kent, 1987; van Dieën et al., 2008; van Dieën and Pijnappels, 2009). At small curb heights, a heel landing is preferred, because toe landings are accompanied with higher effort (i.e., high ankle moment). When increasing the height of the curb, the probability of a toe landing increases, in older adults even more so than in their younger counterparts (van Dieën and Pijnappels, 2009). It has been suggested that a toe landing is preferred for higher curbs, as it allows for more controlled stepping down, since the kinetic energy generated during the step down stays within controllable limits (Buckley et al., 2008; van Dieën et al., 2008). Thus, stepping down a curb using the toe-landing strategy is thought to be safer than stepping down using a heel-landing strategy, at the cost of efficiency (i.e., higher joint moments and a loss of gait speed).

By relating the type of landing chosen to one's actual ability, we can determine whether the selected strategy is adequate. As strategy selection during anticipated stepping down entails a trade off between safety and efficiency, the actual ability measure should quantify the ability to be “safe” (minimizing balance threat) for fair comparison. This can be determined by the ability to regain balance after unexpected stepping down (van Dieën et al., 2007). Due to a sudden drop in walking surface, potential energy is quickly transformed to kinetic energy, and a large amount of kinetic energy should be dissipated to avoid balance loss (van Dieën et al., 2007). Another advantage of this paradigm is that the outcome is unlikely to be affected by one's perception of physical abilities, as there is no time for planning a motor strategy.

We aimed to investigate whether older adults select motor strategies in accordance with their actual physical ability, by comparing the selected strategy during an expected stepping down with the ability to recover from unexpected stepping down. The strategy selection during expected stepping down reflects participants' perceived ability, while kinetic energy reduction during the unexpected step reflects participants' actual ability. As we expect some individuals to select more and others to select less appropriate motor strategies, we hypothesized a moderate positive correlation between the ability to recover after an unexpected step down and the height at which subjects switched between heel landing and toe landing.

## Methods

### Participants

Twenty-one healthy older adults participated in this study (for descriptives see Table 1); however, due to a technical error during data collection, data of one participant were excluded from further analysis. Participants were included if they reported no neurological or muscular impairments, were able to continuously walk for 10 minutes, had a mini-mental state examination (MMSE) of 25 or higher, and did not take medication, which could affect their gait stability. This study was carried out in accordance with the recommendations of “De Nederlandse Gedragscode Wetenschapsbeoefening,” Association of Universities in the Netherlands. The protocol was approved by the “Vaste Commissie Wetenschap en Ethiek” (# VCWE 2016-129). All participants gave written informed consent in accordance with the Declaration of Helsinki.

**Table 1:** Participant descriptives.

**Descriptives:**				
Age		71	[7.25]	years
Females		7	(33%)	persons
Medication (≥4 different medicine)		3	[14%]	persons
Self-reported physical activity		1240	[309]	mins./week
Fallers (≥ 2 falls in the past year)		7	(33%)	persons
Falls in the past year		1	[1]	falls
MMSE		29	[2]	points
FES-I		18	[3.25]	points
Body weight		68.7	[12.3]	kg
Body height		1.69	[0.12]	m
Grip strength		284	[96.9]	N
Max. knee-extension torque		79	[4.6]	Nm

*Prevalence with percentage of sample, or median values and interquartile range (IQR), the latter is indicated by the square brackets (i.e., median [IQR])*.

### Protocol

**Figure 1:**
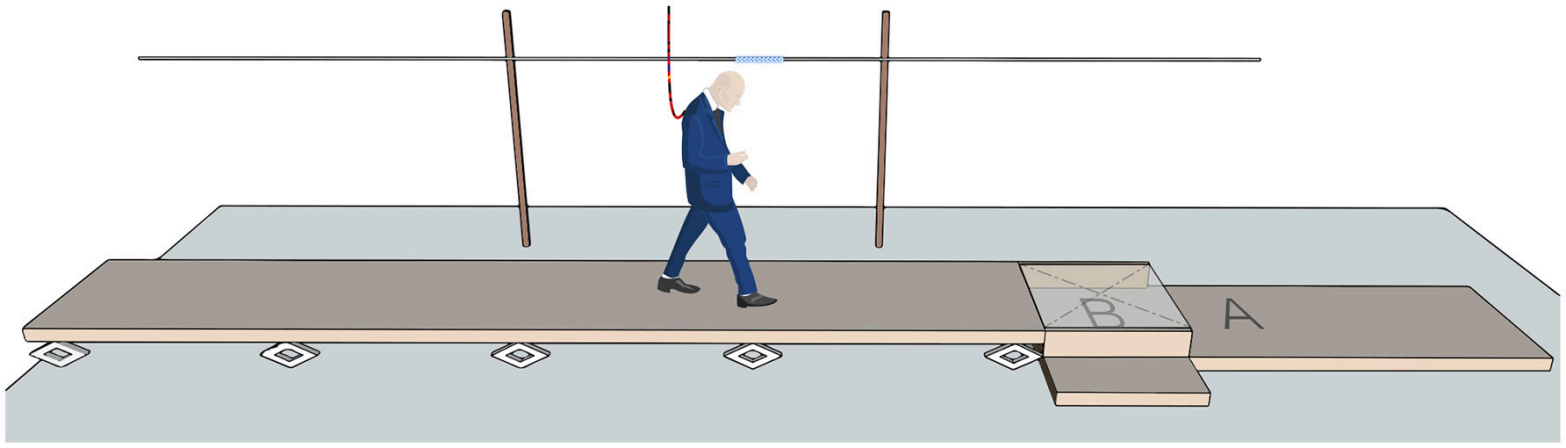
Illustration of the experimental set up. Participants were asked to step down at the end of the platform (landing position indicated by A). The step height was adjustable with jacks from 0.025 to 0.15 m. During the unexpected stepping down condition participants stepped down at position B, while they believed that they would step down, as instructed, at position A, with a height difference of 0.1 m. Participants were secured by a safety harness fixed with climbing ropes to the ceiling. We instructed the participant to adopt the walking speed of 1.1 m/s, as indicated by a LED strip alongside the platform.

Participants were asked to walk over a 7 by 1.2 m platform, adopting the same speed as a set of light emitting diodes, which moved at a speed of 1.1 m per second alongside the platform at eye height of the participant (Figure 1). Participants were asked to step down at the edge of the platform while maintaining the indicated walking speed as much as possible. The height difference was adjustable and the participant was subjected to six different step heights (0.025, 0.05, 0.075, 0.10, 0.125, and 0.15 m). A 1 by 1 m custom-made force-plate was placed behind the height difference, so participants stepped down on top of this force plate (Figure 1). The participant first executed a 0.05 m step down; the landing strategies of six trials were registered and based on the resulting strategies, the platform was either lowered or raised to a new height. We continued varying the height until a height was reached where all six step downs were heel landings (lower bound) or toe landings (higher bound), and heights between the lower and upper bound were registered.

A multinomial logistic regression was fitted to the landing strategy data, and the height at which the chance that a toe landing was used equaled the chance that a heel landing was used (i.e., P_toe_ = P_heel_ = 50%) was defined as the critical height (h_crit_).

Subsequently, for the unexpected stepping down trials, participants were again instructed to step down at the middle of the platform, but instead stepped down earlier than they expected (Figure 1). The height difference was kept at 0.1 m and we informed the participants that they could experience unexpected stepping down during some of the next 15 trials. Three unexpected step downs were randomly assigned to one of these trials. The trial before the first unexpected step was considered as a normal walking condition. Behind the platform, a piece of black cloth spanned two bars, which were tensed by springs and kept in place by magnets, such that the cloth seemed to be walkable and part of the platform. When one of the foot markers crossed the actual height difference, it triggered the magnets to switch off, causing the cloth to quickly drop 0.1 m. This manipulation ensured a heel landing in the unexpected trials. In the other trials, the cloth covered a solid wooden platform of 0.1 m height. During all walking conditions, participants wore a safety harness attached by ropes to a railway mounted to the ceiling, to assure that the participant would not hit the floor if a fall occurred.

### Data acquisition and analyses

**Figure 2:**
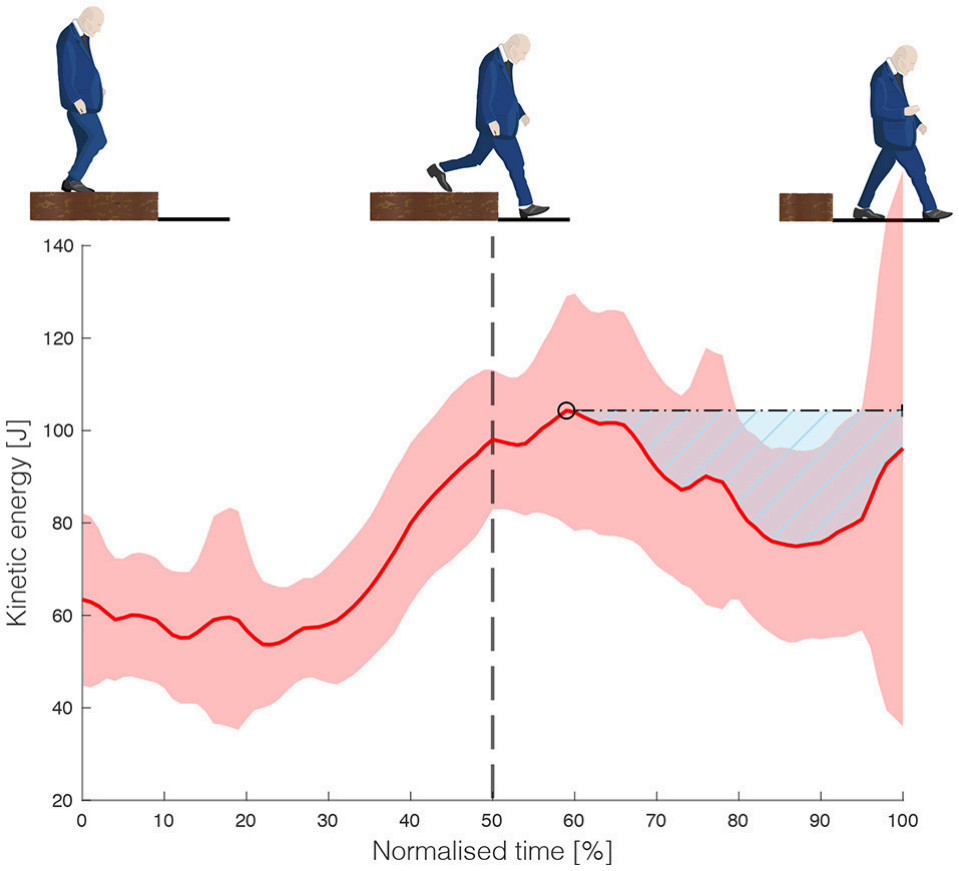
Kinetic energy, time normalized from mid stance, via leading leg landing to trailing leg landing. The ability to recover from unexpected stepping down is defined by the area above the curve (marked by the masked area in the figure) between the peak in kinetic energy after landing and the trailing foot landing.

The positions of 12 infrared light emitting clusters of three markers were captured by three camera arrays (OptoTrak, Northern Digital Inc., Ontario, Canada), to measure full-body kinematics. A kinematic model with 12 linked segments was fitted to the kinematic data, resulting in kinematic trajectories without missing data (van den Bogert et al., 2013). The segments orientations of the most distal segments of which cluster markers were not visible during data collection for less than 30 consecutive samples, were interpolated using a spherical linear interpolation (Dam et al., 1998). The total kinetic energy (i.e., rotational and translational kinetic energy of all segments) and the mechanical work of the leading-leg joints were calculated for (I) expected and (II) unexpected stepping down and for (III) normal walking conditions (the last trial prior to the unexpected stepping down). The kinetic energy during the unexpected stepping down was time normalized from the mid stance before stepping down to the leading-leg landing, and from this event again to first trailing-leg step after landing. Gait events were determined using the kinematic data and afterwards visually checked to ensure correct timing of these events. The peak in the time-normalized kinetic energy was identified and the kinetic energy reduction after the occurrence of this peak and before the trailing foot landing was determined to represent the ability to recover from an unexpected step down (see Figure 2 for illustration). Additionally, the body's angular momentum was calculated (reported in the Supplementary Material) as an alternative measure for safety in terms of balance control.

### Paradigm validity

The validity of our paradigm was evaluated by examining the kinetic energy and mechanical work based on three criteria. First, we expect no difference in kinetic energy between normal walking and unexpected stepping down before the expected heel contact (i.e., the position where the participant believed they would land). Second, similar to the findings of van Dieën et al. (2008), we expected a higher reduction in normalized kinetic energy and higher negative mechanical work done by the ankle during toe landings as compared to heel landings. Only the heel and toe landings that were observed within one step height in the expected condition were analyzed for this within-subject comparison. This comparison was based on a subset of the sample (*N* = 18), as two participants were very consistent in their strategy selection and did not switch strategies within any given step height. Finally, a higher kinetic energy was expected during unexpected stepping down compared to expected stepping down. Since unexpected stepping down always resulted in a heel landing, only heel-landing strategies during the expected stepping from a height of 0.1 m were selected for this comparison. It should be noted that not all participants performed a heel-landing at the 0.1 m step height, hence this comparison was made only based on a subset of the sample (*N* = 11).

### Statistical analysis

Statistical parametric mapping (SPM, Friston et al., 2007; Pataky et al., 2013) was used to test the three assumptions for construct validity of the paradigm. SPM two-tailed paired t-tests were used to identify differences between conditions, and resulting in the weighted magnitude of the differences [referred to as SPM(t)] for the entire time series. To test the null hypothesis, the smoothness of the time series was estimated and a threshold was calculated using Random Field Theory (Adler and Taylor, 2007) and α = 5%, implying that 5% of random but equally smooth curves would exceed this threshold. The null hypothesis is rejected at the instances the SPM(t) value exceeds the threshold. Further technical notes about this procedure can be found elsewhere (Friston et al., 1994). SPM analyses were performed using the spm1d package^1^. Next, the association between h_crit_ as the perceived stepping down ability and the actual ability to recover from an unexpected step down was evaluated using linear regression. Data analyses were performed using custom-made Matlab software (The Mathworks Inc., Natick, MA, RRID:SCR_01622).

## Results

Six expected condition trials out of a total of 120 trials needed to be excluded from further kinematic analyses due to unforeseen technical errors in the kinematics.

**Figure 3:**
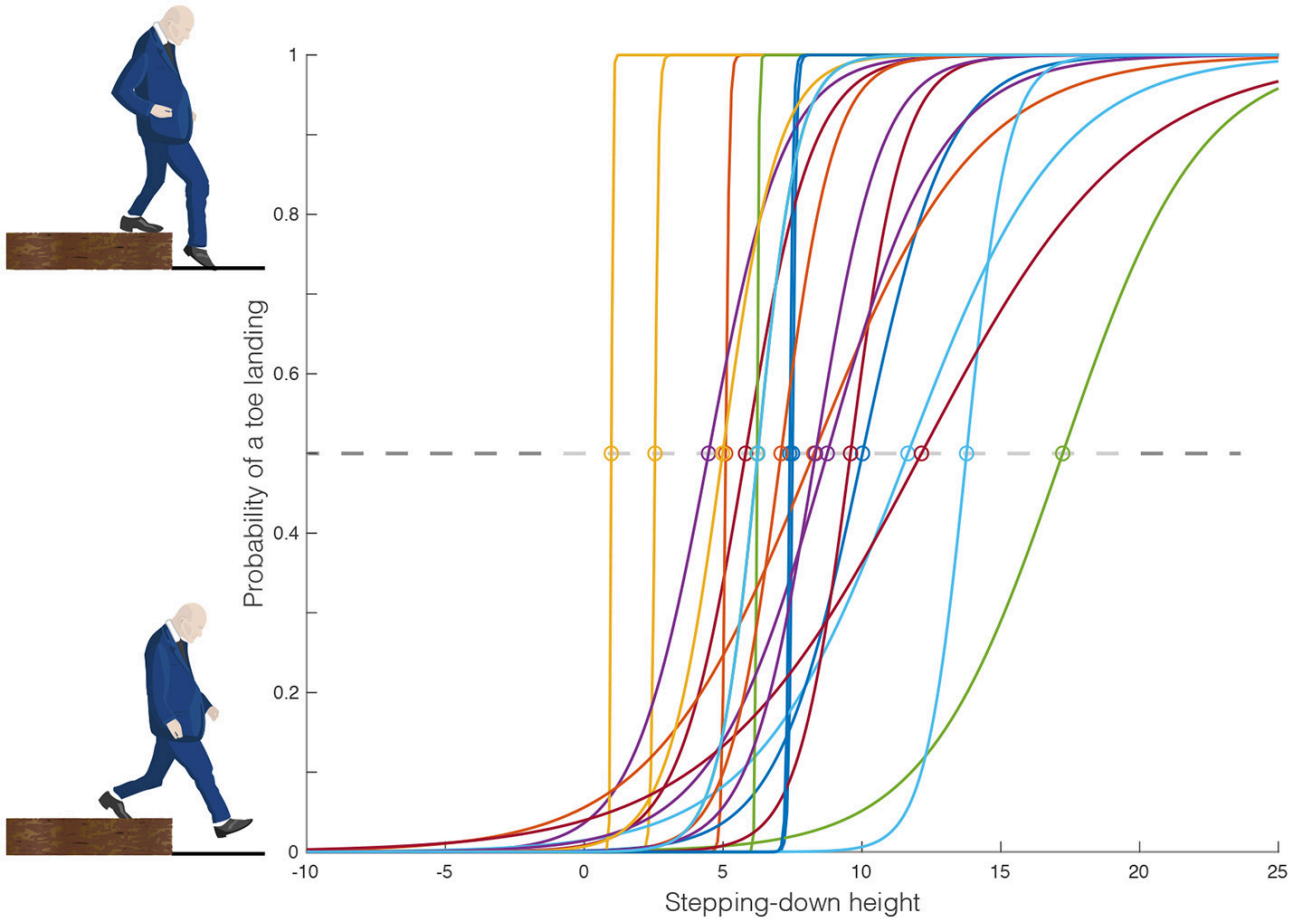
Each participant's stepping-down behavior, determined as the probability of a toe landing by the step height using logistic regression. The individual lines depicts the logistic fits, and the critical switching height was defined as the height at which the probability of a toe landing equaled 0.5.

**Figure 4:**
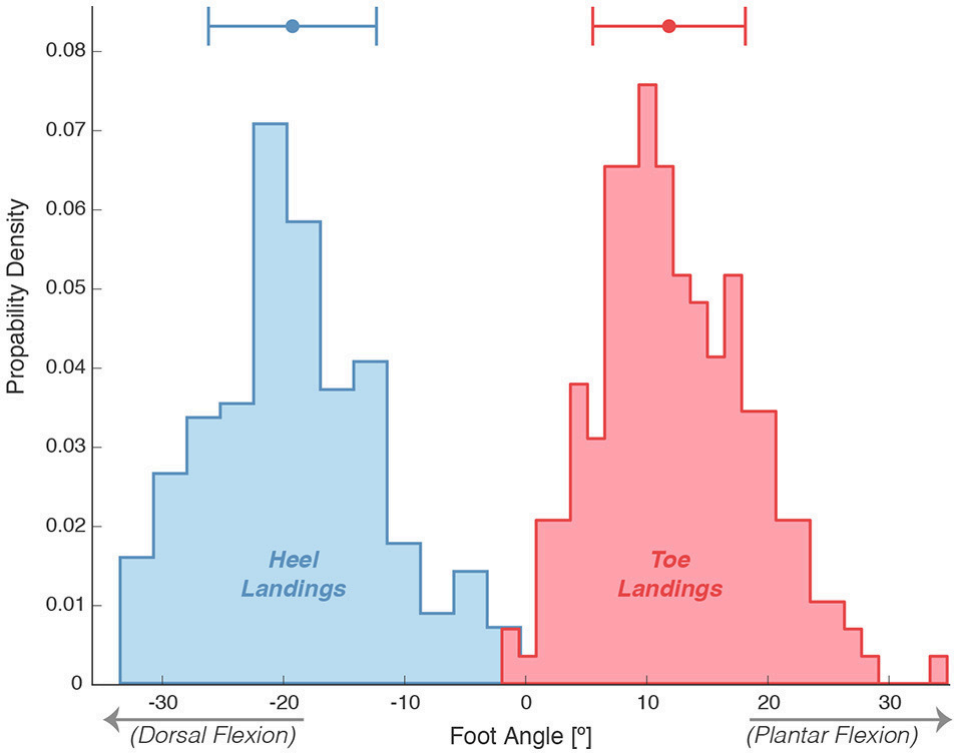
Distributions of foot angles at first foot contact during stepping down. Positive angles are indicative for a plantar flexion orientation of the foot, where negative values represent a more dorsal flexion orientation.

The variability in the participants' critical height, h_crit_, as determined by logistic regression is presented in Figure 3. The logistic regression was fitted using binomial data, Figure 4 displays the foot angle at foot contact during the step down. This data was shaped as a bimodal distribution, which confirms that stepping-down strategy selection is a binary process.

**Figure 5:**
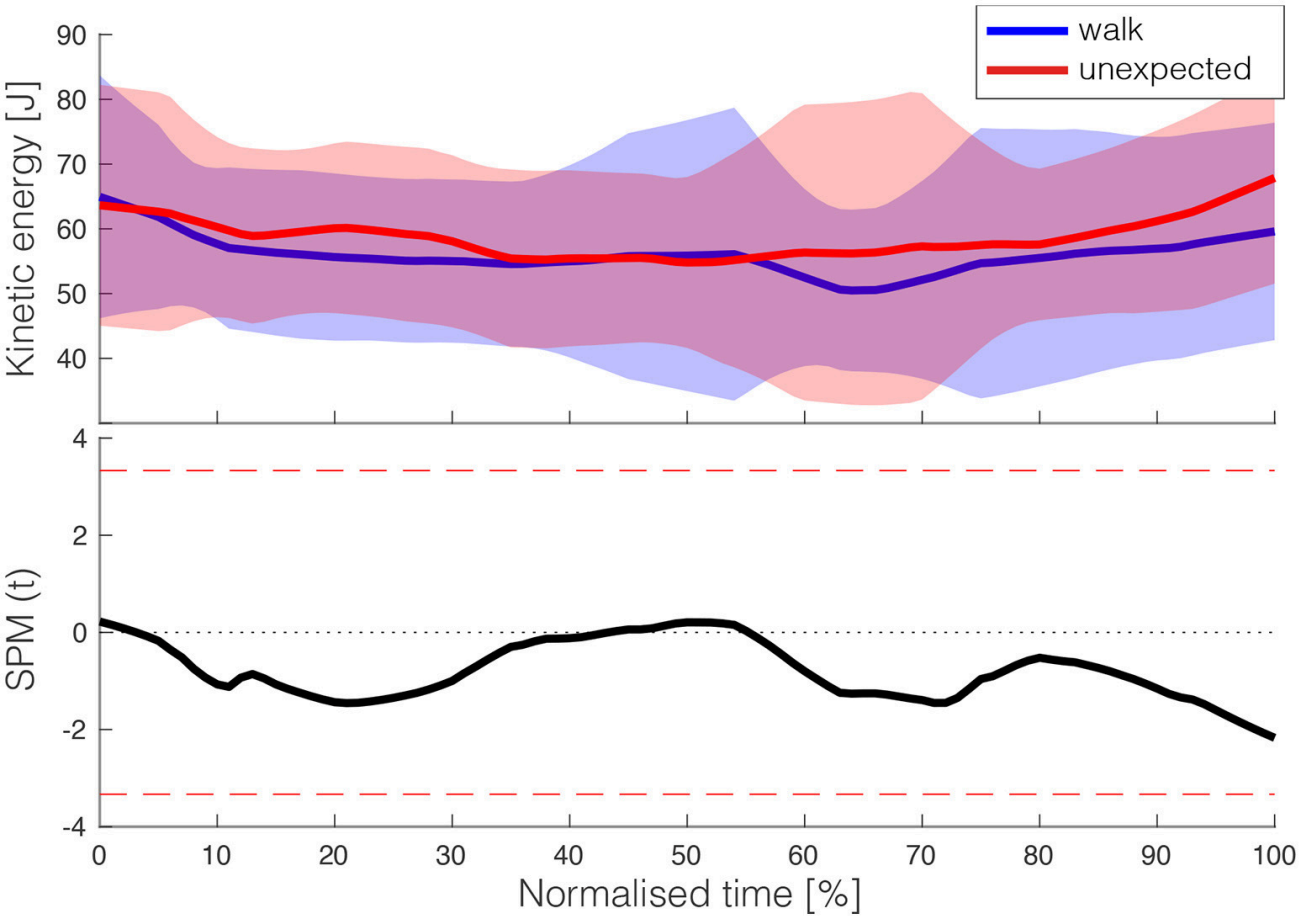
**(Top)** depicts kinetic energy between mid stance and expected foot landing for normal walking and unexpected landing. For normal walking, the expected foot landing coincides with actual foot landing, while in the unexpected condition, it was the instant at which the vertical position of the leading foot crossed the platform level, where participants believed it would touch the ground at this moment in time, while actually this happened a fraction later. **(Bottom)** shows the SPM(t) results, indicating no significant differences between the two conditions.

One participant performed toe landings in every trial. During normal walking, this participant performed heel landings, so we added an additional height of 0 cm and assumed that for this individual those were all heel landings, this led to an h_crit_ of 0.97 cm. The first assumption for validity assessment was met, as the test statistic [SPM(t)] did not exceed the threshold of 3.453 (see Figure 5), confirming that kinetic energy did not differ between unexpected stepping down and normal stepping prior to (expected) landing.

**Figure 6:**
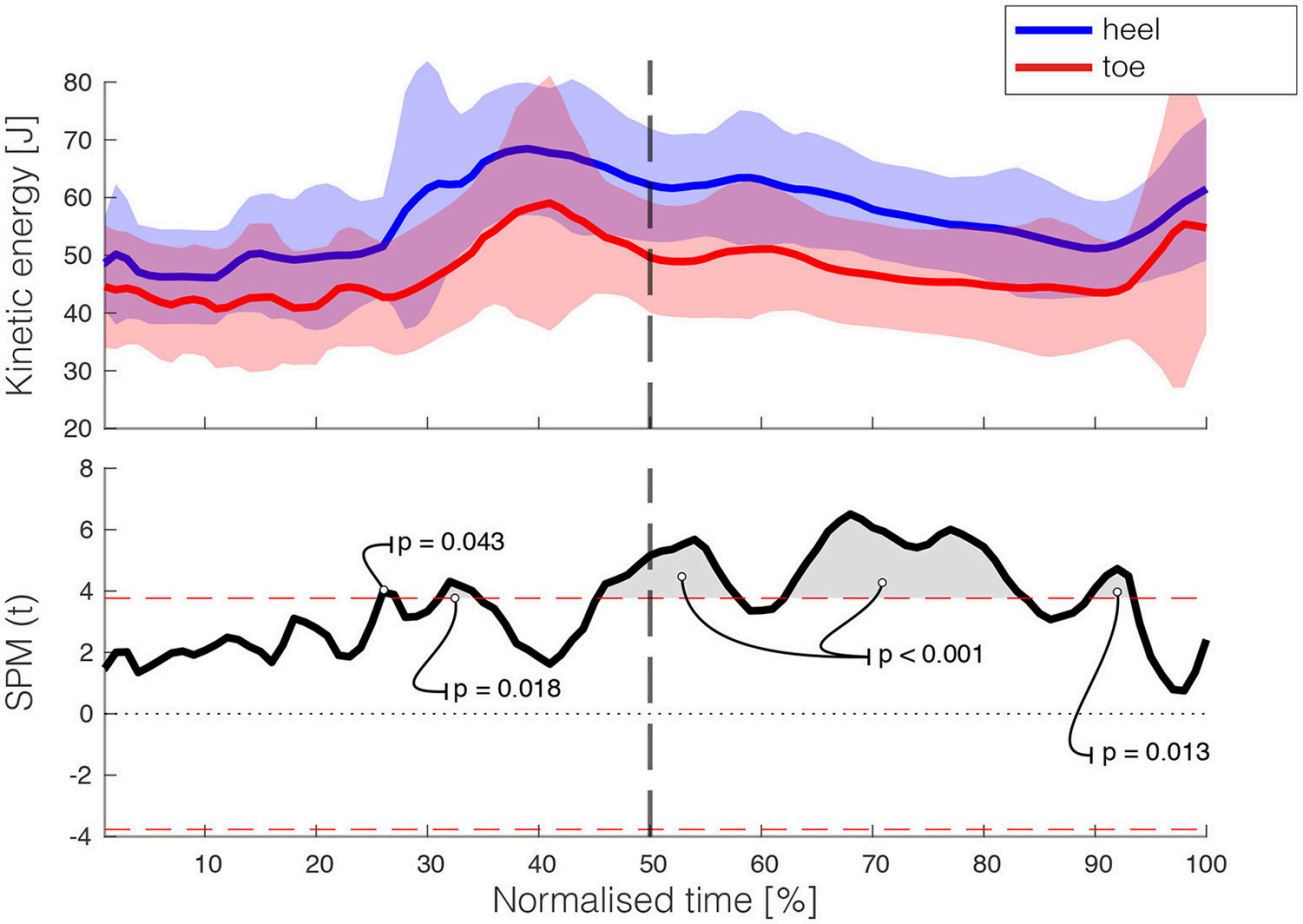
**(Top)** depicts the magnitude of the kinetic energy vector of both the heel and toe landings (± SD is displayed in the gray area). The SPM(t) is shown in the **(Bottom)**, with the gray areas indicating where the difference between the two conditions was significant. The heel landing occurred at 50% of the normalized time (vertical dashed line).

**Figure 7:**
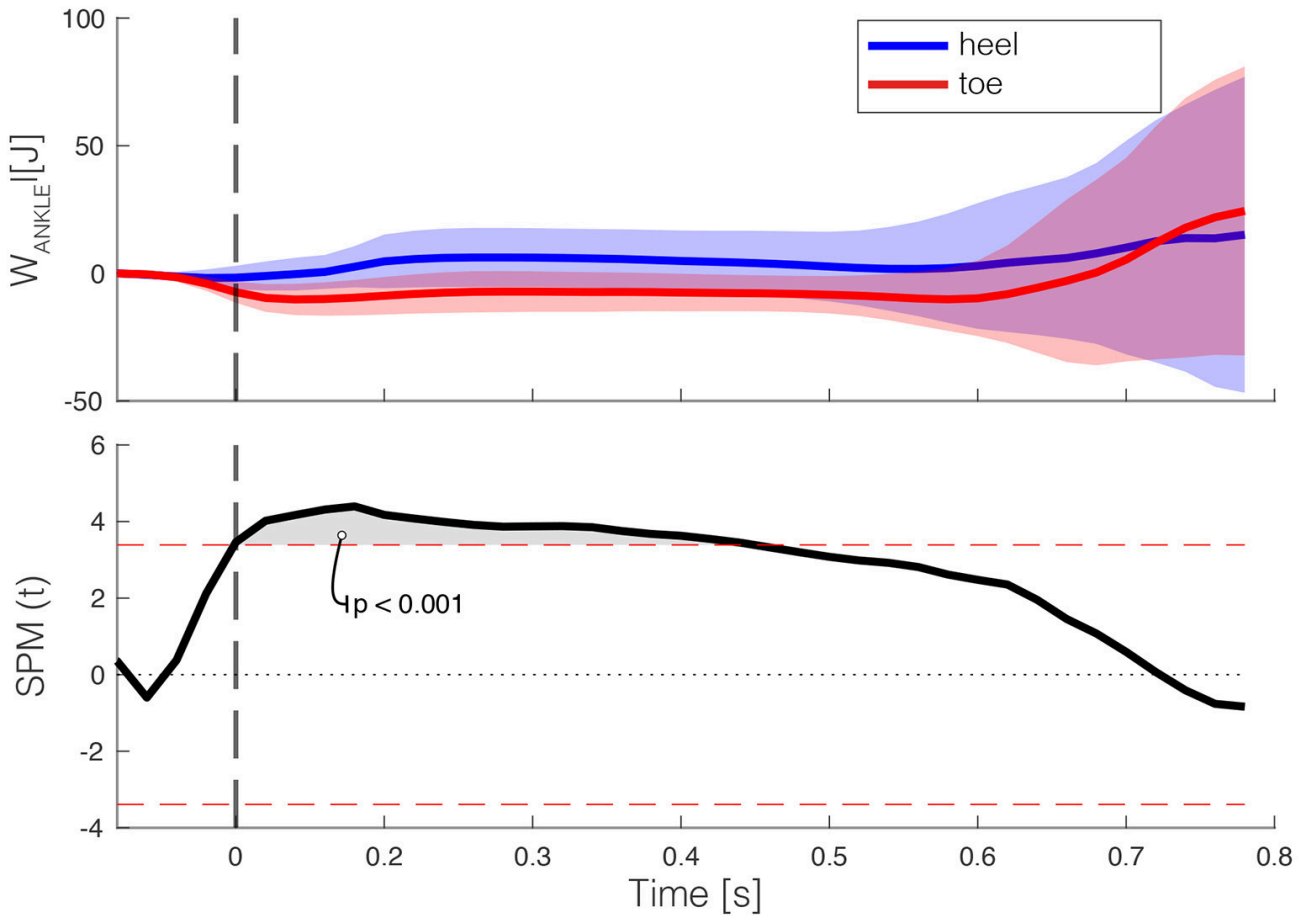
Ankle joint mechanical work in the leading limb **(Top)** averaged over participants during stepping down using a toe-landing strategy (red) and heel-landing strategy (blue). Error bars display ± 1 standard deviation. The SPM(t) is shown in the **(Bottom)**, with the gray areas indicating where the difference between the two conditions was significant. The time series were aligned on foot landing (vertical dashed line).

The second assumption was also met, as more kinetic energy was absorbed during toe landings compared to heel landings (see Figure 6). The SPM(t) exceeded the critical threshold of 3.767 (*p* < 0.001) shortly before landing at the lower platform (at 45% of the normalized time, or 0.08 s before landing), until shortly before the next step (at 93% of the normalized time, or at on average 0.70 s after leading leg landing). The mechanical work done by the ankle was larger after toe landing than after heel landing, as SPM(t) >.389 (*p* <.001) within the first 0.34 s after foot contact (Figure 7).

**Figure 8:**
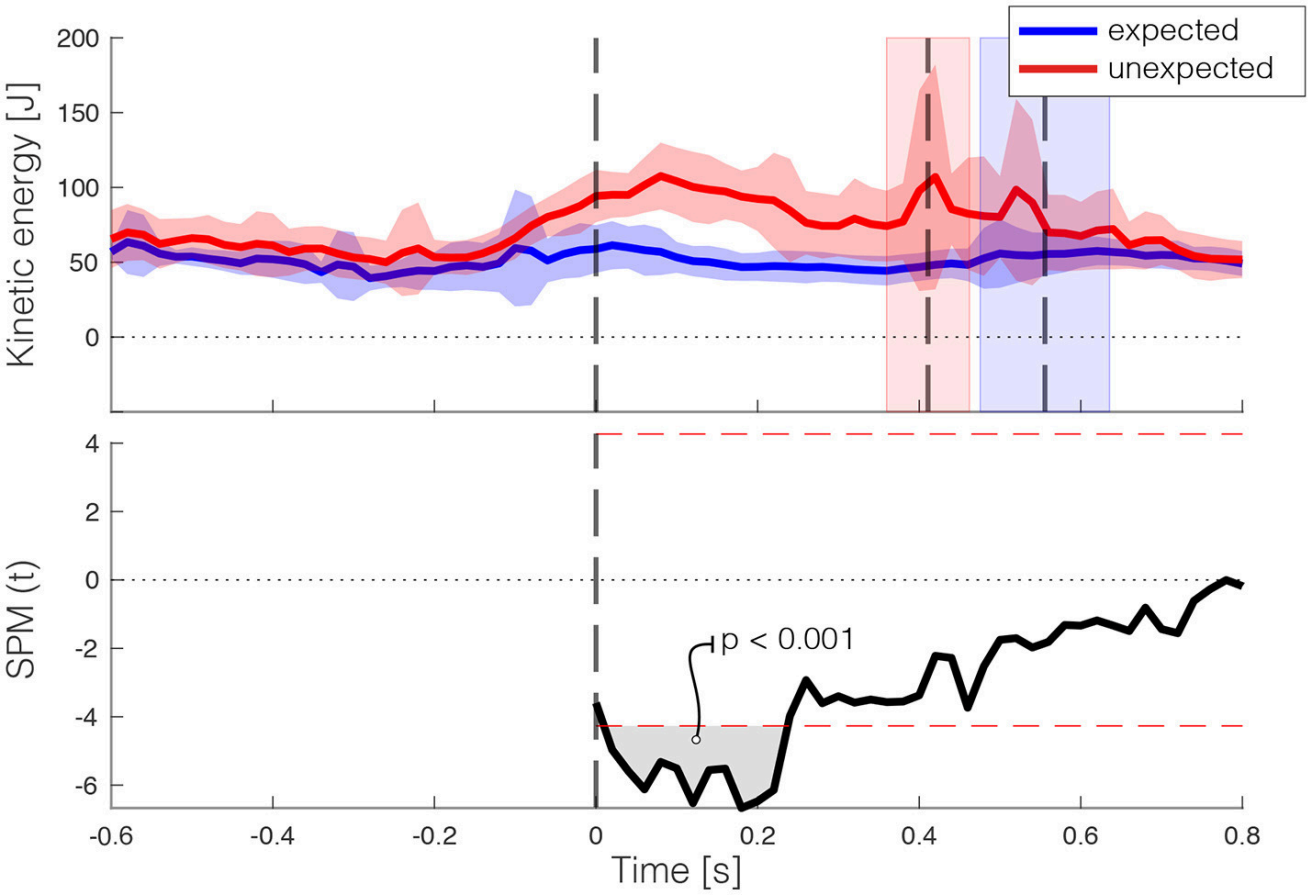
Kinetic energy during step down under two conditions **(Top)**: unexpected stepping down (red) and expected stepping down (blue). The time series of the two conditions were aligned on first foot contact on the lower platform. Note that for fair comparison of expected stepping only heel-landing strategies were considered, as only heel-landing strategies can occur in the unexpected stepping down trial. The vertical line at time 0 displays the moment of leading-foot landing, the trailing-foot landings for both conditions are indicated by the second (unexpected; red) and third (expected; blue) vertical lines. Error bars display ± 1 standard deviation. The SPM(t) is shown in the **(Bottom)**, with the gray areas indicting where the difference between the two conditions was significant.

**Figure 9:**
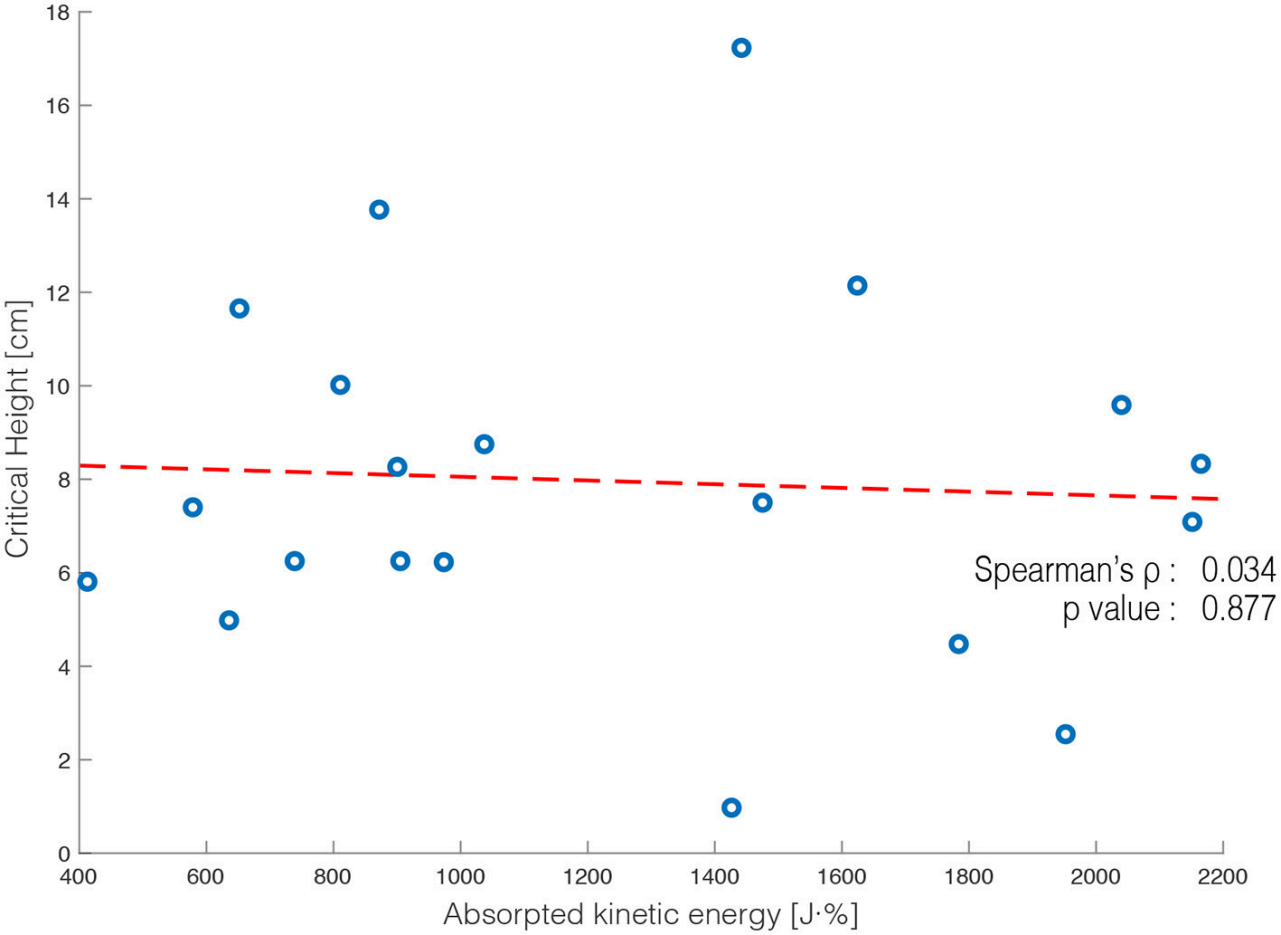
The critical height (h_crit_) and the kinetic energy absorbed after unexpected stepping down displayed for each participant (circles). A best fit line was fitted to the data and the corresponding Spearman's rho and *p*-value are shown.

The third assumption that in unexpected stepping down less kinetic energy is absorbed than in expected stepping down, was also confirmed (Figure 8). In the first 0.2 s after foot contact, the test statistic SPM(t) exceeds the computed threshold of 4.321. Thus, the perturbation effectively increased the kinetic energy in the system after a sudden drop in walking surface, indicating an increase in balance threat. Finally, A non-significant association (ρ = 0.034, *p* = 0.877) was found between h_crit_ (*M* = 7.82 cm, *SD* = 3.91 cm) and the kinetic energy absorbed (*M* = 1228.84 J·%, *SD* = 568.87 J·%) during unexpected stepping down (Figure 9).

## Discussion

The objective of this experiment was to evaluate whether older adults select their movement strategies in line with their physical ability. We expected a moderate positive relation between the switching height and the ability to reduce kinetic energy in (unexpected) stepping down; however, a poor and non-significant association was observed. We offer two arguments that might explain this result.

### Validity of the actual ability

The weak association could be attributed to the kinetic energy measure poorly reflecting one's actual ability. In this study, we assumed that the strategy selection was made based on a trade off between safety (i.e., control of kinetic energy) and efficiency (lower joint moments and maintenance of gait speed). The data showed that, compared to toe landings, the kinetic energy remained larger after heel landing until the onset of the next step of the trailing leg. This result can be seen to confirm our assumed trade off: a too high kinetic energy would be dangerous as it may be indicative of falling, but reducing kinetic energy too much after a step down (as during the toe-landing) may be inefficient, as a certain kinetic energy is required to walk at a given speed.

Alternatively to kinetic energy as an indicator of this threat, angular momentum could possibly be more directly linked to the balance threat imposed (c.f., Pijnappels et al., 2004). However, the gain in sagittal-plane angular momentum during unexpected stepping down appeared only limited (van Dieën et al., 2007, see the Supplementary Material for the evaluation of angular momenta during stepping down in the present data set).

Furthermore, the unexpected perturbation was triggered when a foot marker crossed the curb position. This involved that the timing of the trigger to drop the cloth was not relative to the gait cycle, and hence differed between participants. As the planning of stepping down occurs prior to toe-off of the leading leg–the planning is barely adjusted after toe-off (Timmis et al., 2009)–it is unlikely that participants could have anticipated to the changed circumstances. In support, we did not observe adjustments in kinetic energy prior to the expected landing (Figure 5). Yet, analysis of the angular momentum revealed that before the instant of expected landing the momentum was altered (see Supplementary Material). In the unlikely situation that participants adjusted their behavior, our actual ability measure (i.e., the height of the peak in kinetic energy) could depend on the timing of the perturbation.

It is worth noting that individuals with poor ability to recover from strong perturbations, may be able to handle small perturbations very adequately (c.f., Bruijn et al., 2013). In this study, we determined the participants' ability only once and did not continue until they failed to recover from the perturbation. Given that none of the participants fell, it can be debated how threatening the unexpected step down was. As 12% of falls in older adults occur after erroneous foot placements (e.g., an unexpected step inside a small aperture in the pavement, Berg et al., 1997), we are confident that the manipulation provoked a balance threat that could have led to falling in daily life; however, future research should confirm this.

### Imprecise perception of abilities

The second proposition that could explain the weak association between the strategy selected by participants and the actual ability to recover, is that older adults generally have an imprecise perception of their actual abilities in relation to the task at hand. This is in accordance with previous studies investigating the discrepancy between the perceived and actual step ability (Sakurai et al., 2013, 2016; Kluft et al., 2016, 2017), which found that approximately one-third of older adults either over-or underestimate their abilities, when explicitly asked for their perceived ability. However, in these studies, the perceived and actual ability measures were nevertheless significantly associated, in contrast with the present result.

Our sample consisted of a relative homogenous and fit subgroup of older adults (see Table 1)–enforced by the inclusion criteria–which might have hampered the association between the strategy selected by participants and the actual ability, due to a limited variability in actual abilities. Hence, these findings cannot be extrapolated to the complete population of older adults.

Another source of uncertainty is that aging is associated with a decrease in the ankle plantar flexion torque (Judge et al., 1996). In this study the calf strength was not recorded in the current study. Toe-landing strategies rely heavily on the ability to generate ankle plantar flexion torque; an age-related decrease in the strength of the calf musculature could therefore lead to a decrease of the occurrences of toe landings. On the contrary, overall older adults prefer to step down relatively smaller step heights using toe landings compared to young adults (van Dieën and Pijnappels, 2009).

As the aim of this study was to assess the relations between physical ability and behavioral choice, we did not ask for explicit rating of the participants' perception of their ability. Therefore, we cannot rule out the possibility that the discrepancy between strategy selection and physical ability may have been caused by other factors that could affect strategy selection such as psychological factors (e.g., fear, anxiety, and self-confidence), or physiological factors (e.g., reduced visual acuity Buckley et al., 2005). To develop a full picture of the formation of motor strategy selection, future research focussing on strategy selection and psychological factors is therefore recommended.

## Conclusion

Overall, we did not find a significant association between strategy selection and actual ability. This suggests that the older adults in our study either did not select their movement strategy for stepping down in line with their actual abilities in terms of their ability to absorb kinetic energy after unexpected stepping down, or had an imprecise perception of their actual abilities. Future research should evaluate whether this motor strategy selection is affected by psychological factors, and whether accidental falls in older adults are results of selecting inadequate strategies.

## Author contributions

NK, SB, JvD, and MP contributed to the conception and design of the study. NK build the set up and collected the experimental data. NK and SB developed the code for the data analysis. NK took the lead in writing the manuscript and designed the figures. All authors provided critical feedback and helped shape the research, analysis and manuscript. MP supervised the project. All authors contributed to manuscript revision, read and approved the submitted version.

### Conflict of interest statement

The authors declare that the research was conducted in the absence of any commercial or financial relationships that could be construed as a potential conflict of interest.

## Footnotes

^1^www.spm1d.org
