# Supplementary material for: Do Older Adults Select Appropriate Motor Strategies in a Stepping-Down Paradigm?
Source: Front Physiol. 2018 Oct 12;9:1419. doi: 10.3389/fphys.2018.01419 (PMC6194179; doi:10.3389/fphys.2018.01419)
Supplement: Supplementary file 1 [file Presentation_1.PDF]

Supplementary Material: Do older adults select appropriate motor strategies in a stepping-down paradigm?

Nick Kluft, Sjoerd M. Bruijn, Jaap van Dieën and Mirjam Pijnappels*

*Correspondence:
Mirjam Pijnappels
m.pijnappels@vu.nl

1 SUPPLEMENTARY FIGURES

In addition, and similarly to the kinetic energy analysis, we calculated the angular momentum. The whole body angular momentum was calculated with respect to the center of mass. We were interested in the forward motion, so only the angular momenta in the sagittal plane were used for comparisons.

1.1 Figures: Angular Momentum

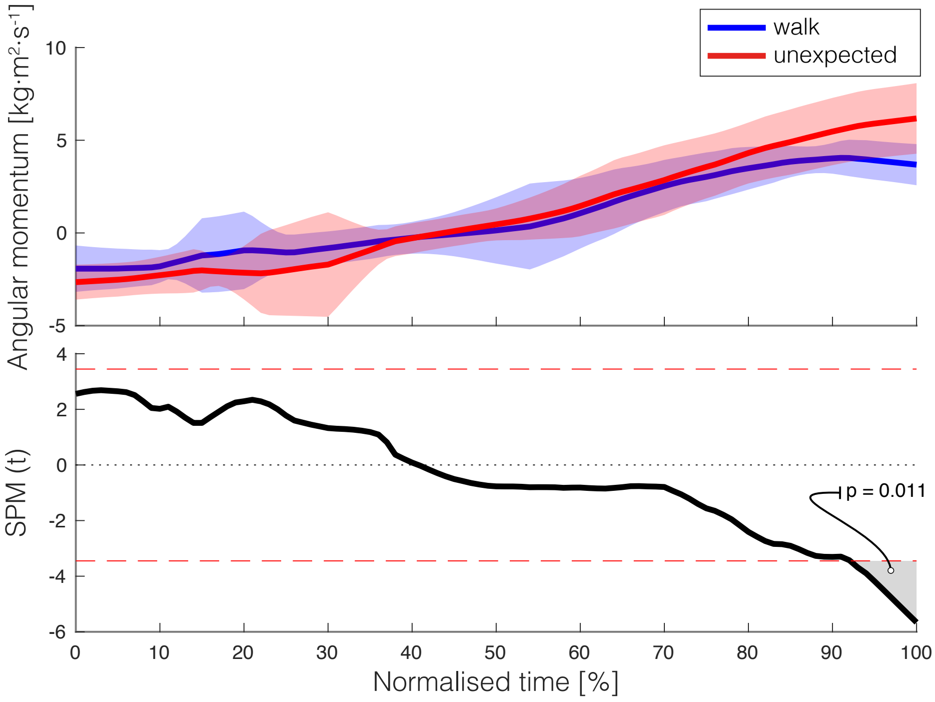

Figure S1. Top panel depicts the angular momentum in the sagittal plane between midstance and expected foot landing for normal walking and unexpected landing. For normal walking, the expected foot landing coincide with actual foot landing, while in the unexpected condition, it was the instant at which the vertical position of the leading foot crossed the platform level, where participants believed it would touch the ground at this moment in time, while actually this happend a fraction later. Error bars display ± 1 SD from the mean trajectory. Bottom panel shows the SPM(t) results, indicating no significant differences between the two conditions. Significant differences are indicated by the grey areas.

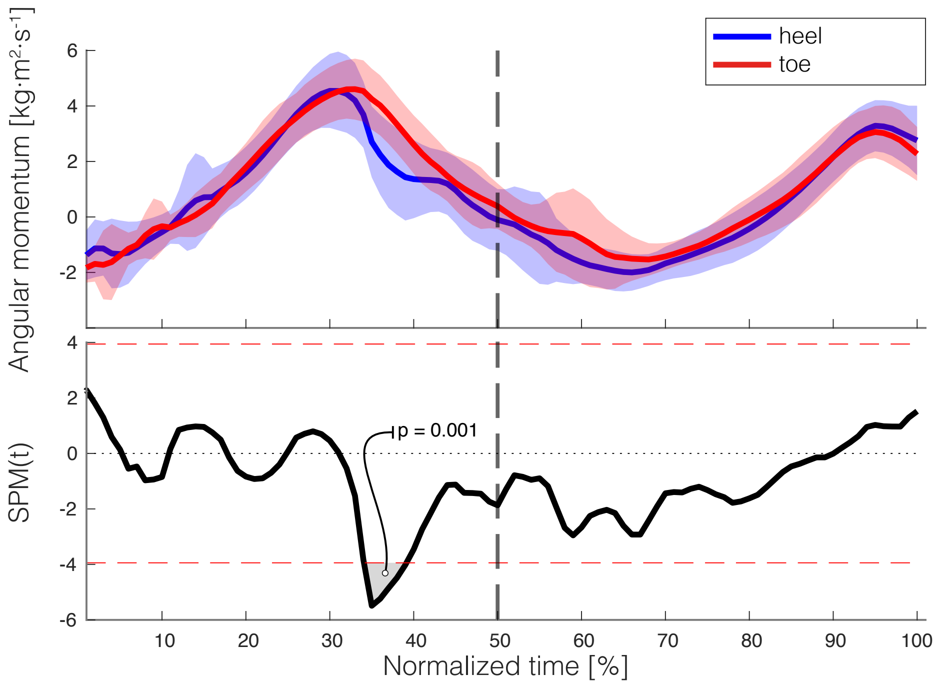

Figure S2. The top panel depicts the magnitude of the angular momentum in the sagittal plane for heel (blue) and toe (red) landings ($\pm$ SD is displayed in the grey area). The SPM(t) is shown in the bottom panel, with the grey areas indicating where the difference between the two conditions was significant. The heel landing occurred at 50% of the normalised time (vertical dashed line).

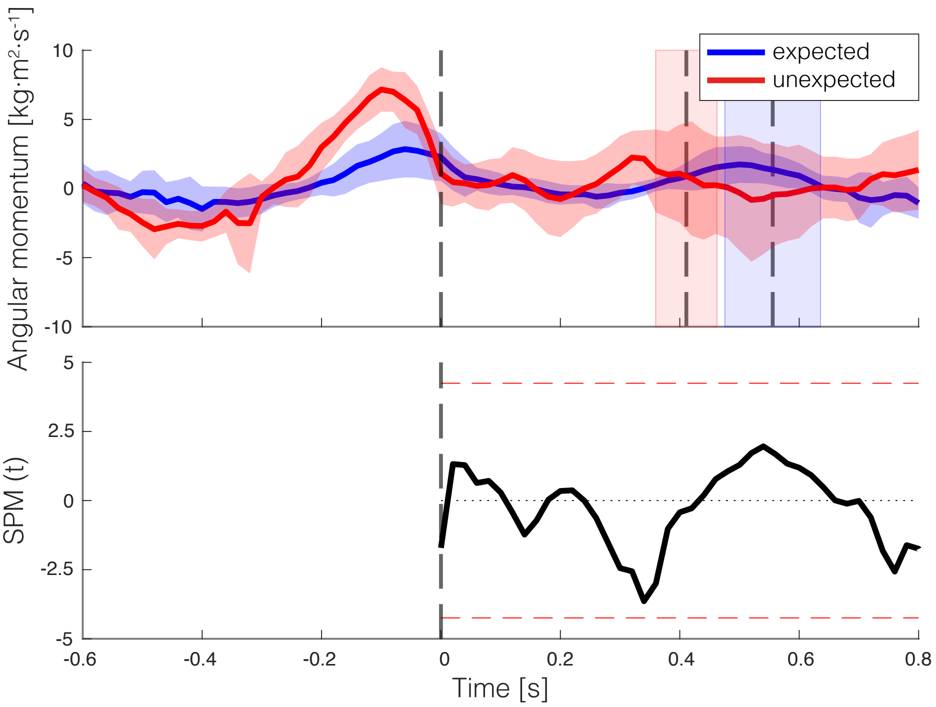

Figure S3. Angular momentum in the sagittal plane of the leading limb during step down under two conditions (top panel): unexpected stepping down (red) and expected stepping down (blue). The time series of the two conditions were aligned on first foot contact on the lower platform. Note that for fair comparison of expected stepping only heel-landing strategies were considered, as only heel-landing strategies can occur in the unexpected stepping down trial. The vertical line at time 0 displays the moment of leading-foot landing, the trailing-foot landings for both conditions are indicated by the second (unexpected; red) and third (expected; blue) vertical lines. Error bars display the mean $\pm$ 1 standard deviation. The SPM(t) is shown in the bottom panel.

2 SUPPLEMENTARY DATA AVAILABILITY

The dataset analysed for the supplementary figures can be found in the Open Science Framework repository (https://osf.io/u6ks4/). All figures in both manuscript and supplementary material can be downloaded from this OSF repository.
